# Comparative Dentinal Tubule Penetration of Three Calcium Silicate-Based Sealers Under Conventional and Sonic-Activated Final Irrigation Protocols: An In Vitro Confocal Microscopy Study

**DOI:** 10.3390/dj14090593

**Published:** 2026-09-14

**Authors:** Israa Ashkar, José Luis Sanz, Leopoldo Forner, Carmen Llena, María Melo

**Affiliations:** Department of Stomatology, Faculty of Medicine and Dentistry, Universitat de València, 46010 Valencia, Spain

**Keywords:** calcium silicate-based sealer, dentinal tubule penetration, confocal laser scanning microscopy, sonic activation, final irrigation, endodontic sealer

## Abstract

**Background**: Successful root canal treatment depends on achieving an effective three-dimensional seal and limiting microbial persistence and reinfection. Dentinal tubule penetration may enhance sealing by increasing sealer–dentin contact and micromechanical retention. This study compared the dentinal tubule penetration of BioRoot RCS, NeoSealer Flo, and TotalFill BC Sealer with AH Plus using the calcium-sensitive fluorophore Fluo-3 and evaluated the effect of sonic activation of final irrigation. **Methods**: 128 extracted human single-rooted teeth were allocated to four sealer groups and subdivided according to final irrigation protocol: non-activated final irrigation (NAFI) or sonic-activated final irrigation (SAFI). Sealers were labeled with 0.1% Fluo-3, and canals were obturated using the single-cone technique. Root sections obtained at 3, 5, and 8 mm from the apex were analyzed by CLSM. Penetration area and depth percentages were calculated and statistically compared (*p <* 0.05). **Results**: All sealers penetrated dentinal tubules. For overall penetration, under NAFI, BioRoot RCS and NeoSealer Flo showed significantly greater penetration area than AH Plus and TotalFill BC Sealer, while BioRoot RCS showed significantly greater penetration depth than AH Plus and TotalFill BC Sealer. Under SAFI, BioRoot RCS and NeoSealer Flo showed significantly greater penetration area than TotalFill BC Sealer, whereas NeoSealer Flo showed significantly greater penetration depth than all other sealers (*p* < 0.05). Sonic activation significantly increased penetration in selected sealers and root thirds; for NeoSealer Flo, penetration depth increased from 37.55% to 49.15% in the coronal third and from 33.10% to 48.73% in the middle third, while AH Plus also showed increased penetration apically and no significant effect was observed for BioRoot RCS. Penetration was generally greatest coronally and lowest apically. **Conclusions**: Dentinal tubule penetration was influenced by sealer type, final irrigation protocol, and root third. Sonic activation improved penetration under specific conditions but did not produce a uniform effect across all sealers or root thirds.

## 1. Introduction

After the introduction of mineral trioxide aggregate (MTA) [1], hydraulic calcium silicate cements became an important part of contemporary endodontics due to their favorable biological behavior, sealing potential, and ability to set under moist conditions [2]. Their subsequent development gave rise to calcium silicate-based sealers (CSBSs), which are now widely used in root canal filling due to their bioactive and physicochemical properties [3].

However, CSBSs should not be considered a uniform material category. Camilleri’s updated classification characterizes dental hydraulic cements according to both material chemistry and clinical use. From a material perspective, hydraulic cements are described by their binder, vehicle, radiopacifier, and additives, which can influence hydration, setting, handling, and biological behavior. Clinically, they are grouped according to their site of use as intracoronal, intraradicular, or extraradicular [4]. Differences in these components and delivery systems can influence hydration, ion release, setting, flow, film thickness, solubility, and dentinal adaptation [4,5,6], potentially affecting penetration into dentinal tubules [7].

Successful root canal filling aims to achieve a three-dimensional seal of the root canal system while limiting pathways for microbial reinfection [8]. Dentinal tubule penetration is commonly evaluated as an indicator of sealer-dentin interaction, although in vitro outcomes vary and may be influenced by material properties and methodological differences among studies [9].

The accuracy of confocal laser scanning microscopy (CLSM) for evaluating sealer penetration also depends on the fluorescent marker used in these studies. Donnermeyer et al. [10] demonstrated that rhodamine B can leach out of sealers and diffuse into dentinal tubules, potentially overestimating the penetration. Therefore, Fluo-3, a calcium-sensitive fluorophore, has been proposed as a more specific alternative for penetration studies of calcium silicate-based materials [9,10]. Although dentinal tubule penetration of CSBSs has been widely investigated, much of the available CLSM evidence relies on rhodamine B, while evidence comparing newer CSBSs using a calcium-sensitive fluorophore and assessing their response to sonic irrigation activation remains limited. NeoSealer Flo was selected as a relatively recently introduced premixed CSBS with a distinct calcium silicate-calcium aluminate formulation and recent studies supporting its physicochemical and bioactive properties [5]. However, evidence regarding its dentinal tubule penetration remains limited. A previous CLSM study evaluated NeoSealer Flo penetration according to obturation technique using rhodamine B [11] but did not compare activated and non-activated final irrigation protocols. Therefore, the effect of sonic activation of final irrigation on NeoSealer Flo dentinal tubule penetration remains insufficiently characterized.

Therefore, the aim of this in vitro study was to compare the dentinal tubule penetration of three CSBSs: BioRoot RCS, NeoSealer Flo, and TotalFill BC Sealer with that of the epoxy resin-based sealer AH Plus using Fluo-3 as a fluorophore under CLSM, and to evaluate the effect of sonic activation of the final irrigation sequence on penetration depth and area. The null hypotheses were that (1) there would be no significant difference in dentinal tubule penetration among the tested sealers and (2) sonic activation of final irrigation would not significantly affect dentinal tubule penetration.

## 2. Materials and Methods

### 2.1. Ethical Approval and Study Protocol

This in vitro study was approved by the Ethics Committee of the University of Valencia (2024-ODON-3284056), with written informed consent obtained for sample use, and was reported according to the PRILE 2021 guidelines [12].

### 2.2. Sample Selection

A total of 128 extracted human single-rooted teeth, including central and lateral incisors, canines, and premolars, with straight roots and closed apices were included. Teeth with previous endodontic treatment, caries, resorption, calcification, fractures, or root curvature were excluded. Root and canal morphology were assessed using periapical radiographs obtained in both buccolingual and mesiodistal projections to evaluate root straightness and the main canal configuration in two planes and to exclude teeth with evident anatomical complexities. However, because conventional periapical radiographs provide a two-dimensional representation of root anatomy, small accessory canals or other anatomical variations not visible in these projections could not be excluded.

The teeth were extracted for clinical reasons unrelated to the study. Patients were informed about the intended research use of the samples and provided written consent for their donation, in accordance with the protocol approved by the Ethics Committee of the University of Valencia (2024-ODON-3284056).

After extraction, teeth were stored in 0.9% saline, disinfected in 2.5% sodium hypochlorite for 48 h, rinsed, and kept again in saline until use, as previously described in another study [13].

Sample size was determined by applying the following formula for comparing two means [14]

N_c_ = N_e_ = 2(Z_α/2_ + Z_β_)^2^ × S^2^/d^2^

Using an SD of 350 µm, α = 0.05, 80% power, and a between-group difference of 250 µm, 32 specimens were allocated to each sealer group and divided equally between irrigation protocols (*n* = 16). AH Plus served as the reference control for comparison with CSBS. To minimize selection bias during allocation, all specimens were stored together in saline in a single opaque container and retrieved individually without visual inspection or selection. Each retrieved specimen was immediately assigned to the next predefined experimental subgroup according to a cyclic allocation sequence, which was repeated until each subgroup contained 16 teeth. This resulted in four sealer groups (N = 32 each: AH Plus, BioRoot RCS, NeoSealer Flo, and TotalFill BC Sealer), with each group divided equally between non-activated final irrigation (NAFI) and sonic-activated final irrigation (SAFI) subgroups (*n* = 16 each).

### 2.3. Root Canal Preparation and Irrigation

Teeth were decoronated to a standardized root length of 15 mm, and working length was established 1 mm short of the apical foramen, as previously described in the literature [15,16]. Canals were prepared with a WaveOne Gold 35/.06 file (Dentsply Sirona, Yverdon-les-Bains, Switzerland) under irrigation with 5 mL of 2.5% NaOCl delivered using a 3 mL Luer-lock syringe and a 27-G closed-end, side-vented needle positioned 1 mm short of the working length, with saline used as an intermediate rinse. For final irrigation, both groups followed the same irrigation sequence and received 3 mL of 17% EDTA for 1 min, followed by saline, 2.5% NaOCl, and then a final saline rinse was performed to remove residual irrigating solution before canal drying and obturation, as previously described [17]. The difference between the two protocols was the use of sonic activation in the SAFI group. In the NAFI group, all solutions were delivered by syringe and needle without activation. In the SAFI group, the EDTA and final NaOCl rinse were each sonically activated for 1 min using the EndoActivator system (Dentsply Maillefer, Yverdon-les-Bains, Switzerland) with a medium 25/.04 tip. Canals were subsequently dried with WaveOne Gold paper points.

### 2.4. Sealers, Fluorophore Labeling, and Obturation

AH Plus^®^ (Dentsply Sirona, Konstanz, Germany) (AHP), used as the epoxy resin-based control; BioRoot™ RCS (Septodont, Saint-Maur-des-Fossés, France) (BR); NeoSealer Flo^®^ (Avalon Biomed, Houston, TX, USA) (NeoF); and TotalFill BC Sealer^®^ (FKG Dentaire, La Chaux-de-Fonds, Switzerland) (TF) were evaluated. The composition and main specifications of the tested sealers are presented in Table 1. Sealers were prepared according to the manufacturers’ instructions and labeled with 0.1% Fluo-3 AM (Sigma-Aldrich, Tokyo, Japan G.K.) for CLSM visualization. They were introduced using a size 2 Lentulo spiral positioned 1 mm short of the working length and operated with an endodontic motor at a controlled low speed of 150 rpm to reduce the risk of instrument fracture. Canals were filled using the single-cone technique, following the manufacturers’ recommendations, with corresponding Medium WaveOne Gold gutta-percha cones. After sealer placement, the corresponding gutta-percha cone was used to complete the obturation, and the quality of the root filling was verified radiographically. Excess gutta-percha was removed with a heated instrument and condensed 1 mm below the canal orifice. Blinding during sealer preparation and placement was not feasible because the tested materials differed in their presentation and handling characteristics, making their identity apparent during use. To minimize technique-related variability, all sealers were prepared according to the manufacturers’ instructions, labeled with 0.1% Fluo-3, placed using the same protocol with Lentulo, and obturated using the single-cone technique.

### 2.5. Sample Sectioning and CLSM Analysis

After root canal filling, CSBS specimens were wrapped in moist gauze, whereas AHP specimens were not. All samples were stored for 72 h at room temperature under 100% humidity to allow sealer setting. Roots were sectioned with a cooled diamond disc to obtain 1 mm thick slices at 3, 5, and 8 mm from the apex, representing the three root thirds, as previously described in the literature [21,22]. Sections were mounted on glass slides, polished under water cooling with fine and superfine Sof-Lex™ XT discs, and stored in the dark until imaging.

**Figure 1:**
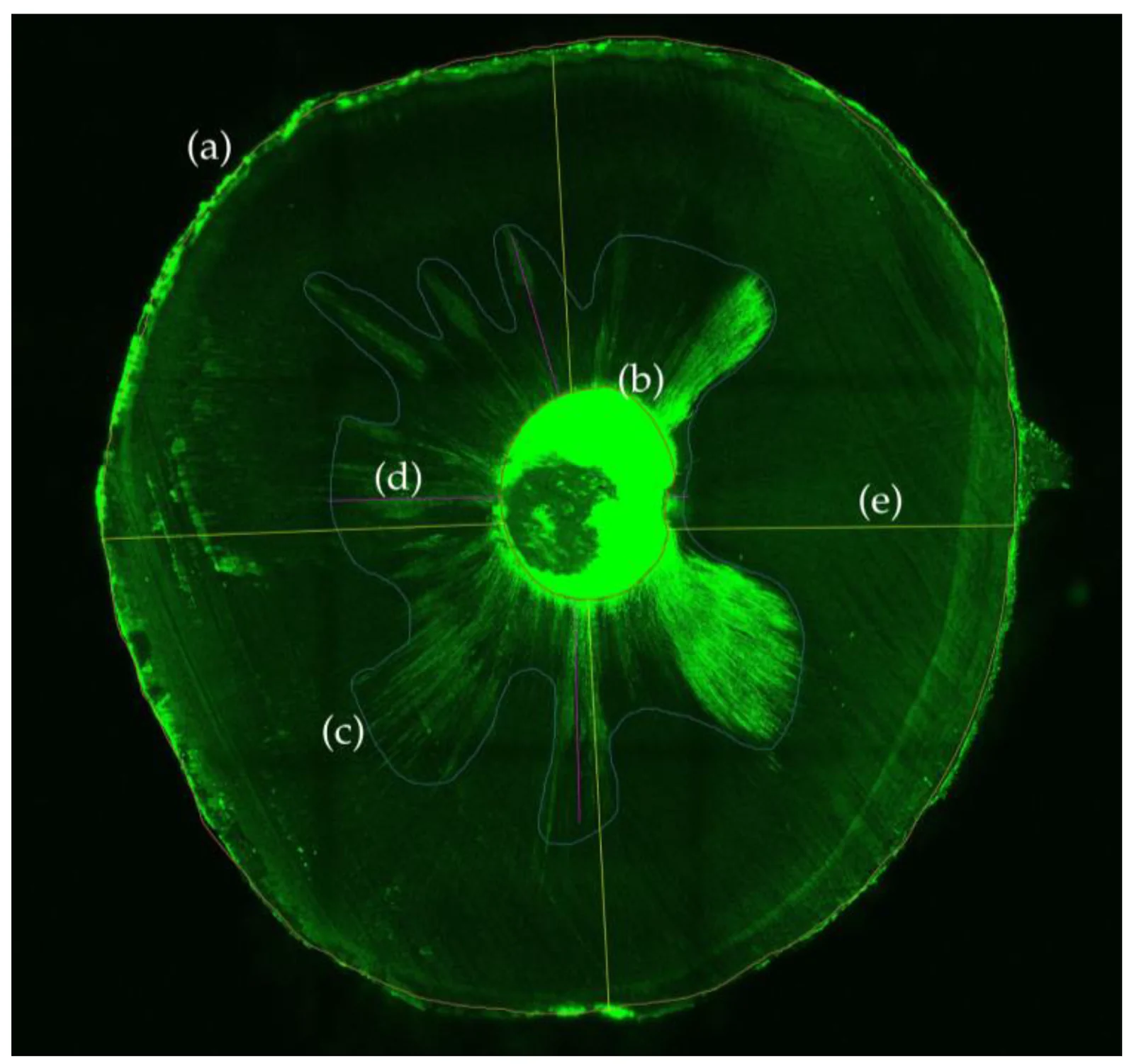
Representative CLSM image showing the measurements recorded for quantitative analysis of sealer penetration: (a) total area; (b) canal area; (c) penetration area; (d) penetration depth; and (e) dentin thickness.

Samples were examined by CLSM (Leica TCS SP8, Leica Microsystems, Wetzlar, Germany) at 10× magnification and analyzed using QuPath 0.5.1 software. All CLSM image analysis and measurements were performed by a single operator. Fluo-3 fluorescence was detected at 506 nm excitation and 519–573 nm emission. Total area, canal area, penetration area, penetration depth, and dentin thickness were recorded, as illustrated in Figure 1. Penetration depth percentage was calculated from the mean maximum penetration depth at four standardized aspects (buccal, lingual, mesial, and distal) relative to the corresponding mean dentin thickness. Penetration area percentage was calculated as the sealer penetration area divided by the total area × 100.

### 2.6. Statistical Analysis

Statistical analysis was performed using SPSS 29.0.1.0 (SPSS Inc., Chicago, IL, USA). Normality and homogeneity of variances were confirmed for each group using the Kolmogorov–Smirnov and Levene tests, respectively. Penetration area and penetration depth percentages were analyzed separately. Comparisons among root thirds were performed using one-way ANOVA followed by Tukey’s post hoc test for pairwise comparisons. Comparisons between NAFI and SAFI within each root third were performed using Student’s *t*-test. Comparisons among the different sealers within each root third were performed using one-way ANOVA followed by Tukey’s post hoc test for pairwise comparisons. Statistical significance was set at *p* < 0.05.

## 3. Results

### 3.1. Overall Penetration

All 128 teeth completed the protocol with no sample loss. Under NAFI, BR and NeoF showed significantly greater penetration area than AHP and TF, while BR showed greater penetration depth than AHP and TF, with NeoF showing intermediate values. Under SAFI, BR and NeoF showed greater penetration area than TF, whereas NeoF showed significantly greater penetration depth than all other sealers (Table 2).

### 3.2. Effect of Sonic Activation

Activation of the final irrigation had either no significant effect on sealer penetration or, when significant; it led to an increase.

**Figure 2:**
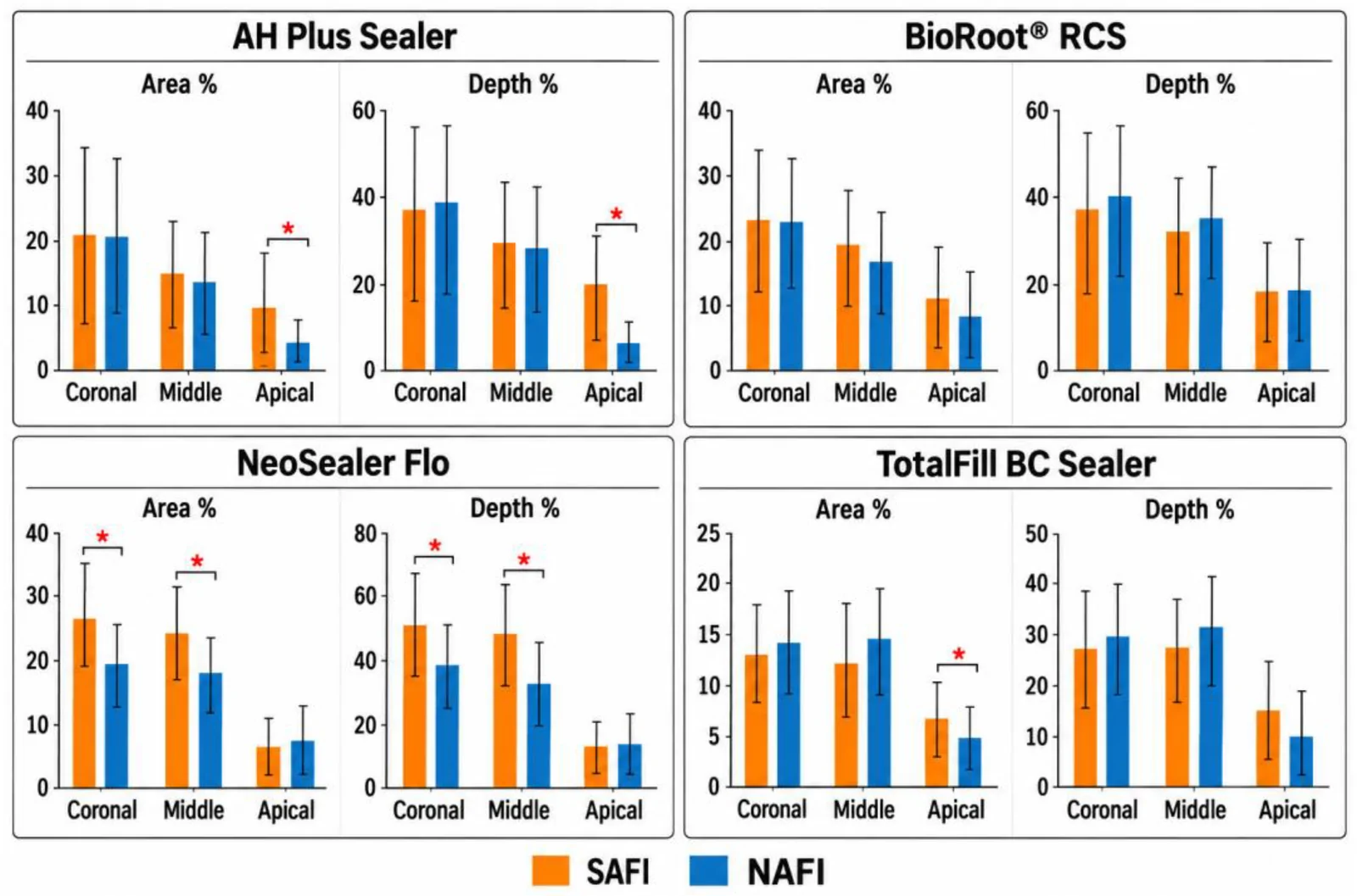
Comparison of dentinal tubule penetration area and depth between NAFI and SAFI for each tested sealer. Values are presented as mean ± SD (*n* = 16 per sealer for each irrigation protocol). Asterisks indicate statistically significant differences between irrigation protocols within the same sealer and root third (*p* < 0.05).

The effect of sonic activation depended on both the sealer and the root third. For AHP, SAFI significantly increased both penetration area and depth in the apical third (*p* < 0.05), with no significant changes in the coronal or middle thirds. BR showed no significant differences between irrigation protocols for either parameter. NeoF showed significant increases in both penetration area and depth in the coronal and middle thirds, whereas no significant effect was observed apically (*p* < 0.05). For TF, SAFI significantly increased penetration area only in the apical third, with no other significant changes in penetration area or depth (Figure 2).

Exact *p* values for comparisons between NAFI and SAFI within each sealer and root third are provided in Supplementary Table S2.

### 3.3. Penetration Across Root Thirds

**Figure 3:**
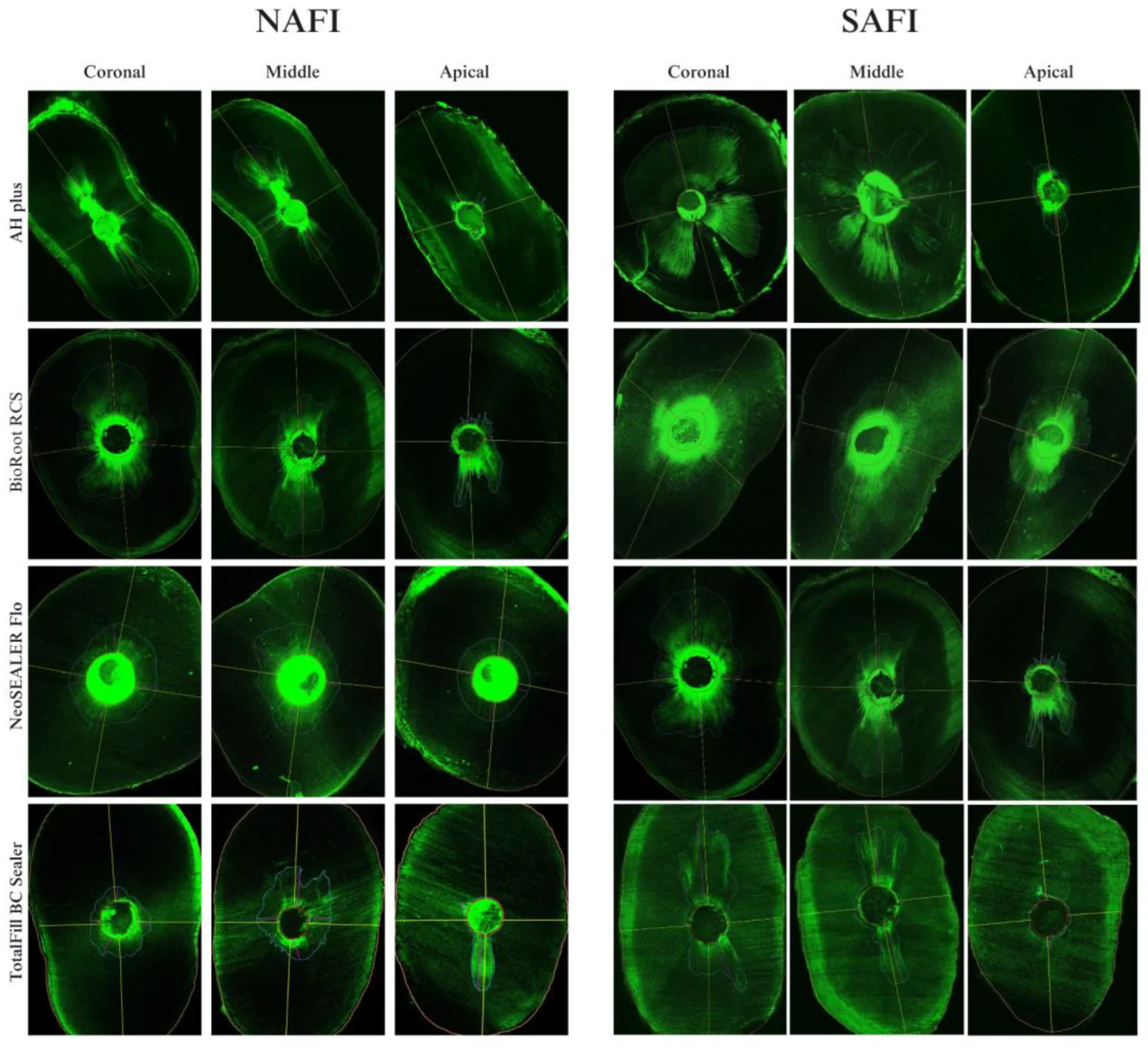
Representative CLSM images showing dentinal tubule penetration of the tested sealers at each third of the root canal in the NAFI and SAFI subgroups. Images were acquired at ×10 magnification. Fluorescence indicates sealer penetration, visualized by Fluo-3 dye (excitation wavelength: 506 nm; emission range: 519–573 nm).

Figure 3 shows representative CLSM images of sealer penetration across root thirds under NAFI and SAFI. Descriptive values by root third are presented in Table 3. Across all sealers and irrigation protocols, the apical third showed significantly smaller penetration area and depth than both the coronal and middle thirds (*p* < 0.05; Supplementary Table S1).

Exact comparisons among sealers within each root third are provided in Supplementary Tables S3 and S4 for NAFI and SAFI, respectively.

## 4. Discussion

The present study rejected both null hypotheses. Sealer type significantly influenced dentinal tubule penetration, while irrigation activation affected penetration only in specific sealers and root thirds. Under NAFI, BR showed the highest overall penetration, whereas NeoF performed best under SAFI.

Fluorochrome selection is important in CLSM studies evaluating CSBSs. Although rhodamine B has been widely used [23,24,25,26], recent studies indicate that rhodamine B is inadequate to be used with CSBS [10]. Its high water-solubility and affinity for moisture could cause rhodamine B to leach out of the sealer matrix and diffuse into dentin independently of the sealer itself, resulting in fluorescence signals in areas not actually infiltrated by the sealer, overestimating apparent penetration depth, and confounding actual material infiltration with dye migration [27,28]. To reduce these artifacts, our study used Fluo-3, a calcium-binding fluorescent indicator that remains non-fluorescent until it binds to free Ca^2+^ ions. Once bound, Fluo-3 emits fluorescence, linking the signal to the sealer’s calcium phase rather than to dye diffusion. This improves specificity, as only regions with released calcium, where the sealer is present, show signal, while non-infiltrated areas remain dark. Coronas et al. [13] demonstrated the feasibility of this approach in dentinal tubule penetration studies, showing that mixing Fluo-3 with a CSBS allowed clear CLSM visualization of sealer penetration while minimizing misleading fluorescence in non-infiltrated zones. In addition, another study [28] compared rhodamine B and Fluo-3 for labeling TF sealer and found that rhodamine B produced higher “penetration” values and greater inter-sample variability, whereas Fluo-3 showed more conservative penetration estimates. Therefore, the use of a different fluorophore in the present study may have contributed to differences between our findings and those of similar sealer penetration studies that used rhodamine B.

However, an important methodological consideration is the incorporation of a fluorophore into the sealer formulation. Although fluorescent labeling is necessary for CLSM visualization, it introduces an additional component that is not part of the manufacturer’s original formulation and may therefore influence material behavior. This concern may be particularly relevant when the fluorophore is incorporated in liquid form. Previous research using 0.1% rhodamine B has shown that fluorophore incorporation can modify physicochemical properties such as flow and setting time, with the effect depending on the sealer and the form in which the dye is added [29]. However, the effect of Fluo-3 incorporation on sealer physicochemical properties has not been specifically established. Since all sealers in the present study were mixed with 0.1% Fluo-3, a potential effect on their flow or setting behavior cannot be excluded.

Among the tested specimens, variations in root morphology were evident, including an anisotropic sealer distribution consistent with the butterfly effect. In these roots, dentinal tubule density and sealer penetration appear to be greater buccolingually than mesiodistally [30,31]. This anisotropic distribution may therefore partly explain the directional differences in sealer penetration observed in the present study. This pattern has also been previously associated with direction-dependent dentinal sclerosis and with the predominantly buccolingual orientation of apical cracks and vertical root fractures [32].

### 4.1. NAFI Subgroup

Under NAFI, BR and NeoF showed the greatest penetration area, while BR also showed the greatest penetration depth. Previous studies on BR have reported conflicting findings, with some showing favorable penetration [33,34,35] and others lower penetration than other CSBSs [16,36,37,38]. These discrepancies may reflect methodological differences among studies, including the sealers evaluated, fluorochrome selection, canal preparation, filling technique, and penetration assessment. Unlike studies based mainly on maximum penetration depth, the present study evaluated both percentage penetration depth at standardized points and overall penetrated area.

### 4.2. SAFI Subgroup

Under SAFI, NeoF showed the greatest overall penetration, while BR also performed well and TF showed the lowest values. NeoF is a relatively new sealer, and its formulation differs from TotalFill/EndoSequence BC, including a lower silicate content and the presence of tricalcium aluminate [5]. Similar to other hydraulic silicate-based cements, NeoF releases calcium ions, enabling interaction with tissue fluids and the subsequent formation of an apatite layer on its surface [39]. A previous study reported higher NeoF penetration values than those observed here, although both studies showed greater penetration in the coronal and middle thirds than apically [11]. The higher values previously reported may partly reflect the use of rhodamine B rather than Fluo-3.

In general, sonic activation showed a selective rather than uniform effect on sealer penetration, producing significant increases in both penetration area and depth for AH Plus in the apical third and for NeoF in the coronal and middle thirds, as well as an increase in penetration area for TF in the apical third. In contrast, BR showed no significant change between irrigation protocols. This suggests that the influence of EndoActivator depends on both the material and the anatomical level rather than producing a generalized increase in penetration. The improved penetration observed in responsive groups may be related to the enhanced fluid dynamics generated by sonic activation. High-amplitude, low-frequency oscillation produces acoustic streaming and increases shear forces along the canal walls, promoting smear-layer disruption and debris removal [40]. Sonic activation may also improve irrigant penetration and cleaning in canal irregularities, potentially increasing dentinal tubule accessibility [41,42,43].

Previous studies have also reported conflicting results, with some showing increased sealer penetration after EndoActivator use [44,45,46] and others finding no significant difference [47,48]. This variability may be related to differences in canal preparation and irrigation protocols, as well as activation parameters such as tip size and power setting [49,50].

### 4.3. Interpretation of the Present Findings

Direct comparison with previous studies is limited by methodological heterogeneity, including differences in irrigation, filling technique, fluorochrome selection, and penetration assessment. This is especially relevant for BR, whose performance has been inconsistent in the literature. Although its viscosity, particle size, and film thickness could limit tubular penetration [51], these properties alone do not explain the wide variation reported across studies [52].

A more likely explanation lies in methodological and analytical factors. In the present study, Fluo-3 may also have influenced the observed sealer performance. Because its fluorescence depends on Ca^2+^, differences in local calcium availability among CSBSs may influence fluorescence intensity [53,54]. BR has been reported to show higher elemental calcium content than TF and AHP [55], which may have contributed to its greater apparent penetration. In addition, the present study assessed penetration as percentages of dentin thickness and area rather than relying only on absolute depth, reducing the influence of anatomical variability (dentin thickness, tooth size, canal morphology, and sectioning level).

Storage and sealer placement may also have affected the results. The specimens were stored for 72 h in a moist environment after filling, which is common in similar studies [13,56], but may influence hydraulic sealers differently. BR is a powder-liquid sealer in which hydration begins immediately after mixing and is therefore less dependent on external moisture [57,58]. In contrast, premixed sealers such as TF and NeoF depend more on environmental moisture to initiate and complete hydration, making their setting behavior and interfacial adaptation more sensitive to storage conditions [59,60,61,62]. Thus, the wet-gauze protocol may have had minimal effect on BR but a greater effect on premixed sealers. Additionally, all sealers were placed with a Lentulo spiral to standardize delivery after Fluo-3 labeling. Although Lentulo spirals provide effective sealer distribution [63,64], this differs from the syringe delivery typically used for premixed sealers and may have influenced their behavior.

From a clinical perspective, dentinal tubule penetration represents a relevant characteristic of root canal sealers as it increases the contact surface between the filling material and radicular dentin and allows the material to extend into areas beyond the main canal lumen [9,65]. Penetration into dentinal tubules may also create a physical barrier around residual microorganisms, potentially preventing further microbial penetration, and contribute to improved sealer retention and root filling stability [23,66]. Recent evidence has demonstrated positive associations between dentinal tubule penetration and push-out bond strength for some endodontic sealers, supporting the potential relevance of penetration to sealer-dentin interaction [67]. Accordingly, the differences in penetration observed among sealers and irrigation protocols in our study may provide clinically relevant information regarding how material selection and final irrigation procedures influence the interaction and distribution of the sealer within radicular dentin. Nevertheless, greater dentinal tubule penetration is only one aspect of root filling performance and does not by itself predict treatment success, since greater penetration does not necessarily correspond to stronger bonding [68]. Thus, greater dentinal tubule penetration may represent an advantageous interfacial property, but it should be considered alongside other factors affecting treatment outcome.

### 4.4. Strengths, Limitations, and Future Perspectives

This study adds to the limited CLSM evidence on NeoF penetration, and it used Fluo-3, which is considered a more suitable fluorophore than rhodamine B for CSBSs [9]. However, its in vitro design using only straight, single-rooted teeth does not fully reflect clinical conditions, as curved canals and multirooted teeth may show different patterns of sealer penetration. Fluo-3 labeling, Lentulo placement, and moist-gauze storage may also have influenced sealer behavior, particularly for premixed materials. All experimental procedures were performed by a single skilled operator to minimize inter-operator variability. However, blinding during sealer preparation and placement was not feasible because the tested materials differed in their presentation and handling characteristics; therefore, some operator-related bias cannot be excluded. Although greater dentinal tubule penetration may improve sealer–dentin interaction, its clinical significance remains unclear. Further studies under more standardized and clinically representative conditions are needed to determine whether greater penetration is associated with better sealing or clinical outcomes.

## 5. Conclusions

Within the limitations of this in vitro study, all tested sealers demonstrated measurable dentinal tubule penetration under CLSM, with penetration generally decreasing from the coronal to the apical third regardless of the irrigation protocol. Under NAFI, BioRoot RCS showed the highest overall penetration, with BioRoot RCS and NeoSealer Flo demonstrating significantly greater penetration area than AH Plus and TotalFill BC Sealer, while BioRoot RCS also showed significantly greater penetration depth than AH Plus and TotalFill BC Sealer. Under SAFI, NeoSealer Flo showed the highest overall penetration, with NeoSealer Flo and BioRoot RCS demonstrating significantly greater penetration area than TotalFill BC Sealer and NeoSealer Flo showing significantly greater penetration depth than all other sealers. Sonic activation had a selective effect, significantly improving AH Plus penetration in the apical third, NeoSealer Flo penetration in the coronal and middle thirds, and TotalFill BC Sealer penetration area in the apical third, while BioRoot RCS showed no significant change between irrigation protocols. These findings reflect dentinal tubule penetration under the specific in vitro conditions of this study and should not be interpreted as evidence of clinical outcomes.

## Figures and Tables

**Table 1:** Composition and main specifications of the tested sealers.

Sealer	Composition	Presentation Form	Setting Time	Working Time
AH Plus^®^ sealer (Dentsply Sirona, Konstanz, Germany)	Paste A: Bisphenol-A epoxy resin, Bisphenol-F epoxy resin, Calcium tungstate, Zirconium oxide, Silica and Iron oxide pigments. Paste B: Dibenzyldiamine, Aminoadamantane, Tricyclodecane-diamine, Calcium tungstate, Zirconium oxide, Silica and Silicone oil [18]	Two component pastes	8 h	4 h
BioRoot™ RCS (Septodont, Saint-Maur-des-Fossés, France)	Powder: tricalcium silicate, zirconium oxide and povidone. Liquid: aqueous solution of calcium chloride and polycarboxylate [19]	Powder and aqueous solution	Approx. 4 h	Approx. 15 min
NeoSealer Flo^®^ (Avalon Biomed, Houston, TX, USA)	Tricalcium silicate, dicalcium silicate, calcium aluminate, calcium aluminum oxide, tricalcium aluminate, tantalite and calcium sulfate [20]	Premixed, ready-to-use paste in a syringe	11 h ± 1 h	>40 min
TotalFill BC Sealer (FKG Dentaire, La Chaux-de-Fonds, Switzerland)	Zirconium oxide, dicalcium silicate, tricalcium silicate, calcium phosphate monobasic, calcium hydroxide, filler, thickening agents [19]	Premixed, ready-to-use paste in a syringe	approx. 2 h	> 4 h

h: hour; min: minute; Approx: approximately.

**Table 2:** Overall penetration area and depth under both final irrigation methods.

Parameter	Subgroup	AHP	BR	NeoF	TF
Penetration area (%)	NAFI	12.68 ± 6.81 (a)	16.18 ± 3.89 (b)	14.88 ± 2.18 (b)	11.05 ± 2.69 (a)
	SAFI	14.88 ± 9.05 (ab)	18.30 ± 5.74 (b)	19.28 ± 3.73 (b)	10.66 ± 1.86 (a)
Penetration depth (%)	NAFI	22.16 ± 10.00 (a)	31.56 ± 8.88 (b)	27.62 ± 3.03 (ab)	23.58 ± 4.94 (a)
	SAFI	25.99 ± 12.28 (a)	29.48 ± 6.85 (a)	36.68 ± 7.03 (b)	23.15 ± 5.58 (a)

Values are mean ± SD. Different letters within the same row indicate significant differences among sealers (*p* < 0.05).

**Table 3:** Penetration area and depth by root third according to final irrigation protocol.

Parameter	Root Third	AHP	BR	NeoF	TF
NAFI subgroup					
Penetration area (%)	Coronal	20.97 ± 11.42 (ab)	23.51 ± 7.03 (a)	19.32 ± 3.14 (ab)	14.26 ± 5.08 (b)
	Middle	13.48 ± 10.24 (a)	16.49 ± 6.72 (a)	17.97 ± 4.83 (a)	13.93 ± 3.81 (a)
	Apical	3.62 ± 1.00 (c)	8.55 ± 5.20 (a)	7.36 ± 3.70 (ab)	4.97 ± 1.76 (bc)
Penetration depth (%)	Coronal	36.31 ± 16.74 (ab)	40.29 ± 10.43 (a)	37.55 ± 6.58 (ab)	28.96 ± 9.29 (b)
	Middle	24.97 ± 15.20 (b)	35.04 ± 10.71 (a)	33.10 ± 6.26 (ab)	31.21 ± 8.32 (ab)
	Apical	5.23 ± 4.17 (b)	19.37 ± 14.04 (a)	12.21 ± 6.25 (ab)	10.58 ± 5.57 (b)
SAFI subgroup					
Penetration area (%)	Coronal	21.02 ± 15.57 (ab)	23.66 ± 8.36 (a)	26.68 ± 8.77 (a)	13.23 ± 3.21 (b)
	Middle	14.32 ± 10.39 (bc)	19.77 ± 7.25 (ab)	24.78 ± 6.76 (a)	11.87 ± 2.72 (c)
	Apical	9.33 ± 9.73 (a)	11.47 ± 7.07 (a)	6.40 ± 3.76 (a)	6.89 ± 2.45 (a)
Penetration depth (%)	Coronal	34.42 ± 19.85 (b)	36.72 ± 10.36 (ab)	49.15 ± 16.41 (a)	26.65 ± 10.19 (b)
	Middle	25.46 ± 15.58 (b)	32.91 ± 9.44 (b)	48.73 ± 9.67 (a)	26.95 ± 7.61 (b)
	Apical	18.09 ± 13.69 (a)	18.81 ± 10.33 (a)	12.18 ± 6.88 (a)	15.87 ± 9.03 (a)

Values are mean ± SD. Different letters within the same row indicate significant differences among sealers (*p* < 0.05).

## Data Availability

The data supporting the findings of this study are available from the corresponding author upon reasonable request.

## Supplementary Materials

The following supporting information can be downloaded at https://www.mdpi.com/article/10.3390/dj14090593/s1: Table S1: Pairwise comparisons of penetration area and depth among root thirds according to sealer and irrigation protocol; Table S2: Comparison of penetration area and depth between NAFI and SAFI within each sealer and root third; Table S3: Pairwise comparisons among sealers within each root third under non-activated final irrigation (NAFI); Table S4: Pairwise comparisons among sealers within each root third under sonic-activated final irrigation (SAFI).
