# Supplementary material for: Comparative Dentinal Tubule Penetration of Three Calcium Silicate-Based Sealers Under Conventional and Sonic-Activated Final Irrigation Protocols: An In Vitro Confocal Microscopy Study
Source: Dent J (Basel). 2026 Sep 14;14(9):593. doi: 10.3390/dj14090593 (PMC13605060; doi:10.3390/dj14090593)
Supplement: Supplementary file 1 [file dentistry-14-00593-s001.zip › dentistry-4536108-supplementary.pdf]

Supplementary Table S1. Pairwise comparisons of penetration area and depth among root thirds according to sealer and irrigation protocol.

Sealer	Irrigation	Parameter (%)	n	Coronal Mean ± SD (95% CI)	Middle Mean ± SD (95% CI)	Apical Mean ± SD (95% CI)	Coronal vs. Middle *p*	Coronal vs. Apical *p*	Middle vs. Apical *p*
AHP	NAFI	Area	16	20.97 ± 11.42 (14.89–27.06)	13.48 ± 10.24 (8.02–18.93)	3.62 ± 1.00 (3.08–4.15)	0.002	< 0.001	0.001
		Depth	16	36.31 ± 16.74 (27.39–45.23)	24.97 ± 15.20 (16.87–33.07)	5.23 ± 4.17 (3.01–7.46)	0.004	< 0.001	< 0.001
	SAFI	Area	16	21.02 ± 15.57 (12.72–29.31)	14.32 ± 10.39 (8.78–19.86)	9.33 ± 9.73 (4.14–14.51)	0.094	0.016	0.036
		Depth	16	34.42 ± 19.85 (23.85–45.00)	25.46 ± 15.58 (17.16–33.77)	18.09 ± 13.69 (10.79–25.38)	0.125	0.012	0.012
BR	NAFI	Area	16	23.51 ± 7.03 (19.77–27.26)	16.49 ± 6.72 (12.91–20.07)	8.55 ± 5.20 (5.78–11.32)	0.006	< 0.001	0.001
		Depth	16	40.29 ± 10.43 (34.73–45.84)	35.04 ± 10.71 (29.33–40.74)	19.37 ± 14.04 (11.89–26.85)	0.048	< 0.001	< 0.001
	SAFI	Area	16	23.66 ± 8.36 (19.21–28.12)	19.77 ± 7.25 (15.91–23.63)	11.47 ± 7.07 (7.70–15.24)	0.028	< 0.001	0.002
		Depth	16	36.72 ± 10.36 (31.20–42.24)	32.91 ± 9.44 (27.88–37.94)	18.81 ± 10.33 (13.31–24.31)	0.111	< 0.001	0.001
NeoF	NAFI	Area	16	19.32 ± 3.14 (17.65–21.00)	17.97 ± 4.83 (15.40–20.55)	7.36 ± 3.70 (5.39–9.33)	0.317	< 0.001	< 0.001
		Depth	16	37.55 ± 6.58 (34.04–41.05)	33.10 ± 6.26 (29.77–36.44)	12.21 ± 6.25 (8.88–15.54)	0.064	< 0.001	< 0.001
	SAFI	Area	16	26.68 ± 8.77 (22.01–31.35)	24.78 ± 6.76 (21.18–28.38)	6.40 ± 3.76 (4.40–8.40)	0.502	< 0.001	< 0.001
		Depth	16	49.15 ± 16.41 (40.40–57.89)	48.73 ± 9.67 (43.58–53.89)	12.18 ± 6.88 (8.51–15.84)	0.928	< 0.001	< 0.001
TF	NAFI	Area	16	14.26 ± 5.08 (11.55–16.97)	13.93 ± 3.81 (11.90–15.96)	4.97 ± 1.76 (4.03–5.90)	0.749	< 0.001	< 0.001
		Depth	16	28.96 ± 9.29 (24.01–33.91)	31.21 ± 8.32 (26.78–35.64)	10.58 ± 5.57 (7.61–13.55)	0.211	< 0.001	< 0.001
	SAFI	Area	16	13.23 ± 3.21 (11.51–14.94)	11.87 ± 2.72 (10.42–13.31)	6.89 ± 2.45 (5.59–8.20)	0.069	< 0.001	< 0.001
		Depth	16	26.65 ± 10.19 (21.22–32.08)	26.95 ± 7.61 (22.89–31.01)	15.87 ± 9.03 (11.05–20.68)	0.899	0.009	0.003

Values are presented as mean ± SD, with 95% confidence intervals (CI) for the mean shown in parentheses. CI, confidence interval; SD, standard deviation. N = 32 total samples per sealer; n = 16 samples per irrigation subgroup. Exact *p* values represent Tukey's post hoc pairwise comparisons among root thirds within the same sealer and irrigation protocol following one-way ANOVA. Statistical significance was set at $p < 0.05$. NAFI, non-activated final irrigation; SAFI, sonic-activated final irrigation.

Supplementary Table S2. Comparison of penetration area and depth between NAFI and SAFI within each sealer and root third.

Sealer	Parameter (%)	Root third	Irrigation	Mean ± SD	95% CI	*p*
AHP	Area	Coronal	NAFI	20.97 ± 11.42	14.89–27.06	0.993
			SAFI	21.02 ± 15.57	12.72–29.31	
		Middle	NAFI	13.48 ± 10.24	8.02–18.93	0.818
			SAFI	14.32 ± 10.39	8.78–19.86	
		Apical	NAFI	3.62 ± 1.00	3.08–4.15	0.034
			SAFI	9.33 ± 9.73	4.14–14.51	
	Depth	Coronal	NAFI	36.31 ± 16.74	27.39–45.23	0.774
			SAFI	34.42 ± 19.85	23.85–45.00	
		Middle	NAFI	24.97 ± 15.20	16.87–33.07	0.928
			SAFI	25.46 ± 15.58	17.16–33.77	
		Apical	NAFI	5.23 ± 4.17	3.01–7.46	0.002
			SAFI	18.09 ± 13.69	10.79–25.38	
BR	Area	Coronal	NAFI	23.51 ± 7.03	19.77–27.26	0.957
			SAFI	23.66 ± 8.36	19.21–28.12	
		Middle	NAFI	16.49 ± 6.72	12.91–20.07	0.195
			SAFI	19.77 ± 7.25	15.91–23.63	
		Apical	NAFI	8.55 ± 5.20	5.78–11.32	0.194
			SAFI	11.47 ± 7.07	7.70–15.24	
	Depth	Coronal	NAFI	40.29 ± 10.43	34.73–45.84	0.340
			SAFI	36.72 ± 10.36	31.20–42.24	
		Middle	NAFI	35.04 ± 10.71	29.33–40.74	0.556
			SAFI	32.91 ± 9.44	27.88–37.94	
		Apical	NAFI	19.37 ± 14.04	11.89–26.85	0.899
			SAFI	18.81 ± 10.33	13.31–24.31	
NeoF	Area	Coronal	NAFI	19.32 ± 3.14	17.65–21.00	0.005
			SAFI	26.68 ± 8.77	22.01–31.35	
		Middle	NAFI	17.97 ± 4.83	15.40–20.55	0.003
			SAFI	24.78 ± 6.76	21.18–28.38	
		Apical	NAFI	7.36 ± 3.70	5.39–9.33	0.471
			SAFI	6.40 ± 3.76	4.40–8.40	
	Depth	Coronal	NAFI	37.55 ± 6.58	34.04–41.05	0.016
			SAFI	49.15 ± 16.41	40.40–57.89	
		Middle	NAFI	33.10 ± 6.26	29.77–36.44	< 0.001
			SAFI	48.73 ± 9.67	43.58–53.89	
		Apical	NAFI	12.21 ± 6.25	8.88–15.54	0.988
			SAFI	12.18 ± 6.88	8.51–15.84	
TF	Area	Coronal	NAFI	14.26 ± 5.08	11.55–16.97	0.497
			SAFI	13.23 ± 3.21	11.51–14.94	
		Middle	NAFI	13.93 ± 3.81	11.90–15.96	0.088
			SAFI	11.87 ± 2.72	10.42–13.31	
		Apical	NAFI	4.97 ± 1.76	4.03–5.90	0.016
			SAFI	6.89 ± 2.45	5.59–8.20	

Sealer	Parameter (%)	Root third	Irrigation	Mean ± SD	95% CI	*p*
	Depth	Coronal	NAFI	28.96 ± 9.29	24.01–33.91	0.508
			SAFI	26.65 ± 10.19	21.22–32.08	
		Middle	NAFI	31.21 ± 8.32	26.78–35.64	0.141
			SAFI	26.95 ± 7.61	22.89–31.01	
		Apical	NAFI	10.58 ± 5.57	7.61–13.55	0.055
			SAFI	15.87 ± 9.03	11.05–20.68	

Values are presented as mean ± SD, with 95% confidence intervals (CI) for the mean. CI, confidence interval; SD, standard deviation. N = 32 total samples per sealer; n = 16 samples per irrigation subgroup. Exact *p* values represent Student's t-test comparisons between NAFI and SAFI within the same sealer and root third. Statistical significance was set at $p < 0.05$. NAFI, non-activated final irrigation; SAFI, sonic-activated final irrigation.

Supplementary Table S3. Pairwise comparisons among sealers within each root third under non-activated final irrigation (NAFI).

Parameter (%)	Root third	Sealer	Sealers compared with	*p* value
Penetration area	Coronal	AHP	BR	0.762
			NeoF	0.920
			TF	0.057
		BR	AHP	0.762
			NeoF	0.378
			TF	0.004
		NeoF	AHP	0.920
			BR	0.378
			TF	0.218
		TF	AHP	0.057
			BR	0.004
			NeoF	0.218
	Middle	AHP	BR	0.602
			NeoF	0.258
			TF	0.998
		BR	AHP	0.602
			NeoF	0.928
			TF	0.716
		NeoF	AHP	0.258
			BR	0.928
			TF	0.349
		TF	AHP	0.998
			BR	0.716
			NeoF	0.349
	Apical	AHP	BR	0.001
			NeoF	0.013
			TF	0.667
		BR	AHP	0.001
			NeoF	0.745
			TF	0.018
		NeoF	AHP	0.013
			BR	0.745
			TF	0.190
		TF	AHP	0.667
			BR	0.018
			NeoF	0.190
Penetration depth	Coronal	AHP	BR	0.756
			NeoF	0.990
			TF	0.272
		BR	AHP	0.756
			NeoF	0.904
			TF	0.033

Parameter (%)	Root third	Sealer	Sealers compared with	*p* value
		NeoF	AHP	0.990
			BR	0.904
			TF	0.154
		TF	AHP	0.272
			BR	0.033
			NeoF	0.154
	Middle	AHP	BR	0.046
			NeoF	0.147
			TF	0.356
		BR	AHP	0.046
			NeoF	0.955
			TF	0.740
		NeoF	AHP	0.147
			BR	0.955
			TF	0.958
		TF	AHP	0.356
			BR	0.740
			NeoF	0.958
	Apical	AHP	BR	< 0.001
			NeoF	0.100
			TF	0.287
		BR	AHP	< 0.001
			NeoF	0.088
			TF	0.023
		NeoF	AHP	0.100
			BR	0.088
			TF	0.947
		TF	AHP	0.287
			BR	0.023
			NeoF	0.947

Exact *p* values represent Tukey's HSD post hoc pairwise comparisons among sealers within the same root third under NAFI following one-way ANOVA. N = 32 total samples per sealer; n = 16 samples in the NAFI subgroup. Statistical significance was set at $p < 0.05$.

Supplementary Table S4. Pairwise comparisons among sealers within each root third under sonic-activated final irrigation (SAFI).

Parameter (%)	Root third	Sealer	Sealers compared with	*p* value
Penetration area	Coronal	AHP	BR	0.877
Penetration area	Coronal	AHP	NeoF	0.385
Penetration area	Coronal	AHP	TF	0.134
Penetration area	Coronal	BR	AHP	0.877
Penetration area	Coronal	BR	NeoF	0.829
Penetration area	Coronal	BR	TF	0.023
Penetration area	Coronal	NeoF	AHP	0.385
Penetration area	Coronal	NeoF	BR	0.829
Penetration area	Coronal	NeoF	TF	0.002
Penetration area	Coronal	TF	AHP	0.134
Penetration area	Coronal	TF	BR	0.023
Penetration area	Coronal	TF	NeoF	0.002
Penetration area	Middle	AHP	BR	0.162
Penetration area	Middle	AHP	NeoF	0.001
Penetration area	Middle	AHP	TF	0.778
Penetration area	Middle	BR	AHP	0.162
Penetration area	Middle	BR	NeoF	0.223
Penetration area	Middle	BR	TF	0.017
Penetration area	Middle	NeoF	AHP	0.001
Penetration area	Middle	NeoF	BR	0.223
Penetration area	Middle	NeoF	TF	< 0.001
Penetration area	Middle	TF	AHP	0.778
Penetration area	Middle	TF	BR	0.017
Penetration area	Middle	TF	NeoF	< 0.001
Penetration area	Apical	AHP	BR	0.782
Penetration area	Apical	AHP	NeoF	0.573
Penetration area	Apical	AHP	TF	0.707
Penetration area	Apical	BR	AHP	0.782
Penetration area	Apical	BR	NeoF	0.126
Penetration area	Apical	BR	TF	0.193
Penetration area	Apical	NeoF	AHP	0.573
Penetration area	Apical	NeoF	BR	0.126
Penetration area	Apical	NeoF	TF	0.996
Penetration area	Apical	TF	AHP	0.707
Penetration area	Apical	TF	BR	0.193
Penetration area	Apical	TF	NeoF	0.996
Penetration depth	Coronal	AHP	BR	0.971
Penetration depth	Coronal	AHP	NeoF	0.032
Penetration depth	Coronal	AHP	TF	0.451
Penetration depth	Coronal	BR	AHP	0.971
Penetration depth	Coronal	BR	NeoF	0.093
Penetration depth	Coronal	BR	TF	0.228

Parameter (%)	Root third	Sealer	Sealers compared with	*p* value
		NeoF	AHP	0.032
			BR	0.093
			TF	< 0.001
		TF	AHP	0.451
			BR	0.228
			NeoF	< 0.001
	Middle	AHP	BR	0.232
			NeoF	< 0.001
			TF	0.981
		BR	AHP	0.232
			NeoF	0.001
			TF	0.424
		NeoF	AHP	< 0.001
			BR	0.001
			TF	< 0.001
		TF	AHP	0.981
			BR	0.424
			NeoF	< 0.001
	Apical	AHP	BR	0.997
			NeoF	0.373
			TF	0.928
		BR	AHP	0.997
			NeoF	0.272
			TF	0.849
		NeoF	AHP	0.373
			BR	0.272
			TF	0.742
		TF	AHP	0.928
			BR	0.849
			NeoF	0.742

Exact p values represent Tukey's HSD post hoc pairwise comparisons among sealers within the same root third under SAFI following one-way ANOVA. N = 32 total samples per sealer; n = 16 samples in the SAFI subgroup. Statistical significance was set at $p < 0.05$.
